# Ethnic disparities of beverage consumption in infants and children 0–5 years of age; National Health and Nutrition Examination Survey 2011 to 2014

**DOI:** 10.1186/s12937-018-0388-0

**Published:** 2018-08-22

**Authors:** Elieke Demmer, Christopher J. Cifelli, Jenny A. Houchins, Victor L. Fulgoni

**Affiliations:** 1 National Dairy Council, 10255 West Higgins Road, Suite 900, Rosemont, IL 60018-5616 USA; 2 Nutrition Impact, LLC, Battle Creek, MI 49014 USA

**Keywords:** Infants, Children, Beverage, Recommendations, Ethnic disparities, Milk, 100% juice, Sugar sweetened beverage, Nutrient intake

## Abstract

**Background:**

Dietary patterns, including beverage consumption, that are developed during a child’s first few years of life have been shown to impact dietary choices made later in life. Authoritative sources provide beverage recommendations for infants and children; however, it is unclear if these guidelines are followed and what, if any, the differences are among races/ethnicities. The objective of this study was to examine beverage consumption to recommendations among children 0–5 months, 6–11 months, 12–23 months, 2–3 years, and 4–5 years. Additionally, examine how these beverage patterns associate with nutrient intake and to determine if differences exist in beverage consumption among race/ethnic groups (Non-Hispanic White, Non-Hispanic Black, Hispanic, and Asian) in children aged 0–23 months, 2–3 years, and 4–5 years.

**Methods:**

Data from the 2011–2014 National Health and Nutrition Examination Survey (NHANES) for children 0–5 years were analyzed (*n* = 2445). Beverages were classified as follows; milk, 100% juice, diet beverages, sugar sweetened beverages (SSB), and water.

**Results:**

Our results show that regardless of race/ethnicity, dietary recommendation were not always followed. Prior to 6 months, 10% of infants consumed any amount of 100% juice; from 6 to 11 months, 17% of young children were drinking any amount of milk. SSB consumption rapidly increased with age, whereas intake of milk and 100% juice declined after 2 to 3 years of age. Non-Hispanic Black young children consumed the most 100% juice from 2 to 3 years and up, exceeding recommended amounts, and throughout all age groups they consumed the least milk and most SSBs. The decreased intake of nutrient-rich beverages with age was associated with lower intakes of under-consumed nutrients of public health concern. By 4–5 years, 32.7% and 93.8% of children were consuming <EAR for calcium and vitamin D, respectively.

**Conclusions:**

Dietary recommendations for both the introduction of beverages and amounts consumed were not consistently followed for American infants and children 0–5 years. Race/ethnic disparities exist in beverage consumption with Non-Hispanic Black children consuming the least amount of milk and most SSBs. Improving beverage consumption patterns could help improve overall diet quality which directly contributes to overall childhood health.

## Background

Infancy and early childhood are periods of rapid growth and development, which requires adequate consumption of essential macro- and micronutrients. Dietary patterns developed during these early life stages can form the basis for future food and beverage preferences [1, 2], thus potentially impacting early- and later-life development. Beverages provide a substantial amount of kilocalories (kcals) and nutrients to the diets of children [3–5]. While some beverages are nutrient rich, others can be a source of nutrients to limit, such as added sugar. For this reason, the U.S. Department of Agriculture (USDA) and Health and Human Services (HHS) have asked for additional information to better understand beverage patterns among infants and children, as the 2020–2025 Dietary Guidelines for American (DGA) will include recommendations for the 0–2 years age range for the first time [6, 7].

Currently, the American Academy of Pediatrics (AAP) provides recommendations for the introduction of beverages to infants and children. Exclusive breastfeeding is recommended for approximately 6 months and continued breastfeeding for at least 12 months [8]. This recommendation is in part due to evidence suggesting that breastfeeding has a positive impact on the development of a child’s later eating behavior [9, 10]. The AAP also recommends that 100% juice not be introduced to the diet before 1 year of age and, once introduced, consumption should be limited to 4 oz (oz) per day for children 1 to 3 years and 4–6 oz. per day for children 4 to 6 years [11]. These recently updated juice recommendations reflect the research showing that prolonged exposure of the teeth to the sugars in juice is a major contributing factor to dental caries, as well as the fact that fruit juices lack protein and fiber which can predispose children to inappropriate weight gain [11]. Cow’s milk is not recommended for the first year of life as it provides inadequate iron, vitamin E, zinc, and essential fatty acids and excessive amounts of sodium, potassium, chloride, and protein for infants [12]. The introduction of plain whole milk is recommended at 1 year and the AAP suggests 16–24 oz. of milk per day [12]. According to experts, no restriction of fat or cholesterol is recommended for children younger than 2 years, as this is a period of rapid growth and neurologic development with high energy requirements, unless a concern for obesity or family history of cardiovascular disease exists [12]. The Institute of Medicine (IOM) has set Adequate Intakes (AI) for water consumption for 0–5 yrs. While the water AI for infants 7–12 mo of age is 0.8 l/day, it should be noted that all beverages and moisture in foods is included in this AI [13]. See Table 1 for a summary of all beverage recommendations for children 0–5 yrs. of age.

**Table 1:** Beverage recommendations for children

	0–5 months^1^	6–11 months^2^	12–23 months^3^	2–3 years^4^	4–5 years^5^
100% Juice	0	0	4 oz	4 oz	4-6 oz
Water^6^	0	0.8 Liters/day	1.3 Liters/day	1.3 Liters/day	1.7 Liters/day
Milk	0	0	16–24 oz. (whole milk)	2 cups (low fat, fat free)	2.5 cups (low fat, fat free)
Sweetened Beverages	0	0	Treats only, ideally eliminated	Within kcal limit	Within kcal limit

1) *0–5 months* 100% Juice (11, 12, 27), water (12, 27), milk (12, 27), sweetened beverages (12, 14, 27); 2) *6–11 months* 100% Juice (11), water (13), milk (12), sweetened beverages (14); 3) *12–23 months* 100% Juice (11), water (13), milk, (12), sweetened beverages (14); 4) *2–3 years* 100% Juice (11), water (13), milk (3), sweetened beverages (3); 5) *4–5 years* 100% Juice (11), water (13), milk (3), sweetened beverages (3); 6) The adequate intake (AI) is listed for children 6 months to 5 years as recommended by the IOM, these AI’s include water from all beverages and moisture in foods

Starting at 2 years, the 2015–2020 DGA recommends low-fat or fat-free milk, in addition to 100% juice and water, as primary beverages of choice [3]. It should be noted that the DGA recommends no more than half of the fruit group be consumed as 100% juice [3]. The Academy of Nutrition and Dietetics (AND) does not recommend beverages with added sugars for infants and suggests that if toddlers consume sugar sweetened beverages (SSBs), they should be considered treats and potentially eliminated from the diet [14]. If SSB are consumed, overall amounts should be within overall kcal limits [3].

Several recent reports indicate that these recommendations for beverage intake in children are not consistently followed. In a 2004 to 2005 Oregon based population survey of nearly 2000 mothers, more than half (59.9%) of the mothers reported that their 2 year-old child drank a SSB at least 1 day per week [15]. Similarly, among a sample of 1189 parents participating in the 2005–2007 Infant Feeding Practices Study II and who were followed up with when their child was 6 years old, a quarter (25.9%) reported that their children consumed SSBs during infancy [16]. Additionally, these data showed that the prevalence of childhood obesity at 6 years was highest among children who either were introduced to SSBs before 6 months of age or who consumed SSBs more than 3 times per week during age 10–12 months [16]. Additionally, research has shown that higher SSB consumption is associated with decreased milk consumption, which could impact the ability of children to meet nutrient recommendations [17]. These observed beverage consumption patterns among infants and children are of concern as early dietary choices have been shown to influence diet choices made later in life and thus can influence diet quality and nutrient adequacy which are important modifiable risk factors for non-communicable diseases [18].

Few studies have researched beverage consumption in children by race/ethnicity, especially during the first 2 years of life. A trend analysis among children 2 to 11 years from 2003 to 2009 reported a decrease in consumption of 2 or more servings of 100% juice per day among Non-Hispanic White children and an increase among Hispanics [19]. Results from 2007 to 2010 National Health and Nutrition Examination Survey (NHANES) analysis showed that 100% fruit juice consumption was significantly higher among Non-Hispanic Black and Hispanic children compared to Non-Hispanic White children [20]. Marked differences among race/ethnic groups were reported for milk consumption for 2 to 18-year-old children and adolescents based on 2001 to 2004 NHANES data. Only 15% of Non-Hispanic Black children met the recommended servings for milk compared to 42% of Non-Hispanic White children and 49% of Hispanic children [21].

Nutrient rich beverages provide a significant percentage of essential nutrients to the diet of infants and young children [22]. The purpose of this analysis was to examine beverage patterns among American infants and young children (0–5 years); specifically, to determine the proportion of children who consume different beverage types across age groups, examine the impact of these beverage consumption patterns on nutrient adequacy for nutrients of public health concern, and determine if beverage recommendations are followed among varying age and race/ethnic groups.

## Methods

### Subjects

Using data from NHANES 2011–2014, we conducted separate analyses for various young age groups in the U.S. population (males and females combined; total *n* = 2445). Age groups were chosen to align with beverage recommendations from authoritative sources: 0–5 months (*n* = 233), 6–11 months (*n* = 301), 12–23 months (*n* = 397), 2–3 years, (*n* = 839) and 4–5 years (*n* = 675). Analyses were also run to determine if there were any differences among race/ethnic groups; Non-Hispanic White (*n* = 591), Non-Hispanic Black (*n* = 643), Hispanic (*n* = 833), Asians (*n* = 222), and other (*n* = 156). Due to smaller sample sizes for race/ethnic groups, within these race/ethnic analyses the following age breakdowns were used 0–23 months (*n* = 931), 2–3 years, (n = 839) and 4–5 years (*n* = 675) (Table 2). Subjects with other race/ethnicity designations were not studied as they were not representative of the national population. Only subjects with reliable 24-h recall interviews who met minimum criteria as established by the United States Department of Agriculture (USDA) were included in the analyses [22, 23]; additionally, given lack of quantification of intake, those consuming human milk were also excluded (*n* = 260).

**Table 2:** Sample sizes by age and race/ethnicty

Ethnicity	Age Group			
	0-23 Months	2-3 Years	4-5 Years	All
Hispanic	328 (39%)	265 (32%)	240 (29%)	833
Non-Hispanic White	250 (42%)	202 (34%)	139 (24%)	591
Non-Hispanic Black	233 (36%)	242 (38%)	168 (26%)	643
Asian	62 (28%)	80 (36%)	80 (36%)	222
Other	58 (37%)	50 (32%)	48 (31%)	156
All	931 (38%)	839 (34%)	675 (28%)	2,445

### Beverage and nutrient intakes

Day 1 24-h dietary recalls (provided by parents/caretakers) were used to assess intake of specific beverages on any given day using definitions in the USDA food category system used for NHANES [24]. The day 1 recall was collected via an in-person interview and used the validated Automated Multi Pass Method and is the best estimate of consumption on any given day. The beverage groups examined were as follows; 1) 100% Juice: citrus, apple, other fruit, vegetable, baby juice. 2) Water: tap, bottled, flavored, carbonated, enhanced, fortified, baby water. 3) Milk: milk, flavored milk, milk shakes, dairy drinks, milk substitutes. 4) Diet Beverages: diet soft drinks, diet sport drinks, diet energy drinks, other diet drinks. 5) Sugar Sweetened Beverages: soft drinks, fruit drinks, sport drinks, energy drinks, nutritional beverages. 6) Coffee and Tea: coffee, tea. Data representing the percent of children who consumed each beverage (any amount), the amount of beverage consumed, and energy from beverages are presented as mean ± standard error (SE) for each age group. For comparative purposes the percentage of kcals from each beverage was determined along with percent kcals from other foods. The intake of milk and flavored milk based on fat type using USDA defined categories were also determined (i.e., whole, reduced fat, low fat, and nonfat).

### Statistical analyses

The distribution of usual intake including percentiles and percentages meeting cutoffs was estimated using version 2.1 of the National Cancer Institute (NCI) usual intake programs [25]. The estimates are generated with 2 days of dietary data (second day recall was gathered via the telephone 3–10 days after the in-person interview; this second day data are used to help define intra- and inter-person variation) with day 1 dietary weights used in all stages of the estimation process. Balanced repeated replicates (BRR) were used which equal the smallest multiple of 4 that is greater than the number of NHANES strata in the input dataset for the usual intake. A non-response adjustment based on age, gender and race/ethnicity was made to BRR weights for each replication. The covariates used in the usual intake models were dietary recall number (1 or 2), and day of recall coded as weekday or weekend (Friday, Saturday, and Sunday). Nutrients reported included: calcium, iron, magnesium, phosphorus, potassium, zinc and vitamins A, C, and D. Race/ethnicity information came from NHANES demographic files based on responses during the in home questionnaire [26]. SAS 9.2 (SAS Institute, Cary, NC) was used with NHANES design aspects (primary sampling units and strata) and appropriate sampling weights to ensure estimates are nationally representative. Mean amounts, percentages and SEs were determined with “Proc Surveymeans” procedure and differences in means by age and or race/ethnicity were determined by t-tests with *p* < 0.01 as significant.

## Results

### Beverage choices

For the total sample of children, 20.5% of total kcals were from beverages evaluated (289 kcal) and 79.5% of kcals were from food (1100 kcal). The caloric contribution to the overall diet by beverage type were plain milk (10.7%), 100% juice (4.3%), SSBs (3.4%) and flavored milk (2.2%).

Reported beverage consumption on any given day by age among infants and children are presented in Table 3. Regardless of race/ethnicity, over 10% of infants younger than 6 months reported consuming 100% juice and more than 24% consumed small amounts of water despite recommendations to exclusively consume human milk, or in the absence of human milk, iron-fortified infant formula. The IOM recommends no supplementation of water, juice, nonhuman milk, or foods prior to 6 months of age [27]. For infants 6 to 11 months, over 17% consumed milk and 5% drank SSBs. Milk consumption was highest among 1 to 2 years where only 10% did not consume milk. Milk intake declined after the second year of life. More than 12% of 2 to 3-year olds did not consume milk and this number increased to over 17% in 4 to 5 years. While milk consumption decreased with age, consumption of SSBs significantly increased with age. For children 12–23 months, nearly 31% consumed SSBs, which significantly increased to 40% for children 2 to 3 years and to more than 51% for children 4 to 5 years.

**Table 3:** Percent of children consuming different beverage types on a given day, NHANES 2011–2014

	0–5 months (*n* = 233)	6–11 months (*n* = 301)	12–23 months (*n* = 397)	2–3 years (*n* = 839)	4–5 years (*n* = 675)
	Mean ± SEM	Mean ± SEM	Mean ± SEM	Mean ± SEM	Mean ± SEM
100% Juice^1^	10.13^a^ ± 1.96	38.78^b^ ± 3.68	58.23^c^ ± 3.48	53.97^c^ ± 2.37	49.05^c^ ± 3.26
Water^2^	24.69^a^ ± 4.45	61.88^b^ ± 2.92	77.46^c^ ± 2.83	82.18^c^ ± 1.55	79.23^c^ ± 2.30
Milk^3^	0.73^a^ ± 0.54	17.47^b^ ± 3.09	90.86^c^ ± 1.97	87.49^c^ ± 1.47	82.74^c^ ± 2.13
Diet Beverages^4^	0^a^ ± 0	0^a^ ± 0	2.11^ab^ ± 1.08	3.51^b^ ± 0.93	5.07^b^ ± 0.79
Sweetened Beverages^5^	0.66^a^ ± 0.52	5.05^b^ ± 1.00	30.97^c^ ± 3.73	40.19^cd^ ± 3.04	51.32^d^ ± 3.83
Coffee and Tea^6^	0^a^ ± 0	0.55^a^ ± 0.56	7.25^b^ ± 1.70	8.94^b^ ± 1.41	8.37^b^ ± 1.35

Data based on Day 1 dietary recalls

SEM, Standard Error of the Mean. 1) 100% Juice includes: citrus, apple, other fruit, vegetable, baby juice. 2) Water includes: tap, bottled, flavored, carbonated, enhanced, fortified, baby water. 3) Milk includes: milk, flavored milk, milk shakes, dairy drinks, milk substitutes. 4) Diet Beverages includes: diet soft drinks, diet sport drinks, diet energy drinks, other diet drinks. 5) Sugar Sweetened Beverages includes: soft drinks, fruit drinks, sport drinks, energy drinks, nutritional beverages. 6) Coffee and Tea includes: coffee, tea. ^a,b,c,d^ Means sharing the same superscripts are not significantly different across ages, *p* < 0.01

Since milk recommendations vary by milk fat level as children get older, Table 4 presents the amount of milk consumed on any given day by type for each age group. Whole milk was the predominant type consumed by children 2 years and younger. For children 2 to 5 years reduced fat (2%) milk was the predominant type consumed. The mean amount of milk consumed was lower than recommendations for all age groups.

**Table 4:** Amount of milk consumed on a given day by milk-type and age, NHANES 2011–2014

	0–5 months (*n* = 233)	6–11 months (*n* = 301)	12–23 months (*n* = 397)	2–3 years (*n* = 839)	4–5 years (*n* = 675)
	Grams^1^ ± SEM	Grams^1^ ± SEM	Grams^1^ ± SEM	Grams^1^ ± SEM	Grams^1^ ± SEM
Milk, whole	3.15^a^ ± 3.12	53.90^b^ ± 11.80	345.60^c^ ± 26.68	99.12^d^ ± 12.66	57.16^b^ ± 12.68
Milk, reduced fat	0.11^a^ ± 0.11	11.79^b^ ± 3.32	90.49^c^ ± 13.00	150.07^d^ ± 12.46	105.44^c^ ± 8.19
Milk, lowfat	0^a^ ± 0	3.56^ab^ ± 1.98	15.35^bc^ ± 5.14	34.12^c^ ± 6.51	45.46^bc^ ± 15.51
Milk, nonfat	0^a^ ± 0	0.02^a^ ± 0.02	2.25^ab^ ± 1.28	19.58^b^ ± 6.98	17.98^b^ ± 5.74
Flavored Milk, whole	0^a^ ± 0	0.34^a^ ± 0.34	8.23^ab^ ± 4.08	10.79^b^ ± 2.77	20.85^b^ ± 5.88
Flavored Milk, reduced fat	0^a^ ± 0	1.29^a^ ± 1.23	1.07^a^ ± 0.76	31.36^b^ ± 7.71	28.89^b^ ± 5.22
Flavored Milk, lowfat	0^a^ ± 0	0^a^ ± 0	0^a^ ± 0	11.96^b^ ± 3.06	8.53^b^ ± 2.49
Flavored Milk, nonfat	0^a^ ± 0	0^a^ ± 0	0^a^ ± 0	4.97^b^ ± 1.60	8.69^b^ ± 2.66
Dairy Drinks and Substitutes	0^a^ ± 0	5.35^ab^ ± 5.14	22.22^b^ ± 6.44	13.87^b^ ± 3.32	11.22^b^ ± 3.72

Data based on Day 1 dietary recalls

*SEM* Standard Error of the Mean. 1). One cup of milk is equivalent to 244 g. ^a,b,c,d^ Means sharing the same superscripts are not significantly different across ages, *p* < 0.01. Dairy drinks and substitutes includes milk substitutes, milk shakes, and other dairy drinks

The amounts of milk, 100% juice and SSBs consumed on any given day by age and race/ethnicity are presented in Table 5. Milk consumption was highest among Non-Hispanic White children from 0 to 23 months, among Hispanic children from 2 to 3 years, and Asian children consumed the most milk from 4 to 5 years. Milk consumption was consistently the lowest among Non-Hispanic Black children after 2 years of age. Non-Hispanic Black children consumed the most 100% juice during the first 3 years of life, while Asian children consumed significantly less 100% juice from 4 to 5 years than all other races/ethnicities. 100% juice consumption decreased after 3 years across all the race/ethnic groups. Non-Hispanic Black children consumed the highest amount of SSBs among all age groups. In comparison, Asian children consumed the least amount of SSBs during the first 5 years of life.

**Table 5:** Amount of Total Milk, 100% Juice, and Sugar Sweetened Beverages consumed on a given day by age and race/ethnicity, NHANES 2011–2014

	0–23 Months	2–3 Years	4–5 Years
	Mean ± SEM	Mean ± SEM	Mean ± SEM
Milk^1^ (*cups eq.*)			
Non-Hispanic White (*n = 591*)	1.31^a^ ± 0.12	1.50^ab^ ± 0.10	1.30^ab^ ± 0.18
Non-Hispanic Black (*n = 643*)	0.83^b^ ± 0.08	1.14^a^ ± 0.12	0.83^a^ ± 0.10
Hispanic (*n = 833*)	1.21^a^ ± 0.09	1.65^b^ ± 0.10	1.33^b^ ± 0.09
Asian (*n = 222*)	1.06^ab^ ± 0.24	1.61^ab^ ± 0.34	1.43^b^ ± 0.14
100% Juice^2^ *(cups eq.)*			
Non-Hispanic White	0.35 ± 0.06	0.57 ± 0.08	0.47^b^ ± 0.05
Non-Hispanic Black	0.44 ± 0.07	0.80 ± 0.11	0.44^ab^ ± 0.06
Hispanic	0.41 ± 0.04	0.51 ± 0.06	0.47^b^ ± 0.06
Asian	0.31 ± 0.07	0.62 ± 0.09	0.21^a^ ± 0.07
Sugar Sweetened Beverages^3^ *(oz)*			
Non-Hispanic White	1.37^ab^ ± 0.38	3.76^ab^ ± 0.57	5.74^b^ ± 0.92
Non-Hispanic Black	3.54^b^ ± 0.82	5.63^b^ ± 0.70	9.12^b^ ± 1.01
Hispanic	2.28^ab^ ± 0.53	5.21^ab^ ± 0.76	6.79^b^ ± 0.95
Asian	0.63^a^ ± 0.42	2.82^a^ ± 0.75	2.19^a^ ± 0.65

Data based on Day 1 dietary recalls

*SEM* Standard Error of the Mean. 1) Milk includes: milk, flavored milk, milk shakes, dairy drinks, milk substitutes. 2) 100% Juice includes: citrus, apple, other fruit, vegetable, baby juice. 3) Sugar Sweetened Beverages includes: soft drinks, fruit drinks, sport drinks, energy drinks, nutritional beverages. ^a,b^ Means sharing the same superscripts are not significantly different by race/ethnicity within age groups, *p* < 0.01

The kcal contribution of the reported beverage choices on any given day is presented in Table 6. Regardless of race/ethnicity, milk and 100% juice contributed the most kcals for children across most age groups. The contribution of kcals from SSBs significantly increased with age. By 4 to 5 years, SSBs contributed 75 kcals to daily intake compared to 59 kcals from 100% juice.

**Table 6:** Daily kilocalorie (kcal) contribution of beverage choice on a given day by age, NHANES 2011–2014

	0–5 Months *n* = 233			6–11 months *n* = 301			12–23 months *n* = 397			2–3 years *n* = 839			4–5 years *n* = 675		
	Mean		SEM	Mean		SEM	Mean		SEM	Mean		SEM	Mean		SEM
	(% total kcal)			(% total kcal)			(% total kcal)			(% total kcal)			(% total kcal)		
100% Juice^1^	5.44	±	1.38	28.49	±	3.31	65.72	±	7.37	72.20	±	5.10	59.21	±	6.15
	(0.08	±	0.20)	(3.26	±	0.38)	(5.31	±	.60)	(4.94	±	0.35)	(3.66	±	0.38)
Water^2^	0.00	±	0.00	0.00	±	0.00	0.03	±	0.03	0.57	±	0.27	0.57	±	0.40
	(0	±	0)	(0	±	0)	(0	±	0)	(0.04	±	0.02)	(0.04	±	0.02)
Milk^3^	2.22	±	2.15	43.78	±	8.25	281.53	±	14.67	211.15	±	9.80	169.79	±	12.49
	(0.33	±	0.32)	(5.01	±	0.94)	(22.77	±	1.19)	(14.45	±	0.67)	(10.49	±	0.77)
Diet Beverages^4^	0.00	±	0.00	0.00	±	0.00	0.04	±	0.02	0.23	±	0.11	0.32	±	0.20
	(0	±	0)	(0	±	0)	(0	±	0)	(0.02	±	0.01)	(0.02	±	0.01)
Sugar Sweetened Beverages^5^	0.04	±	0.03	2.40	±	0.64	41.79	±	7.29	49.92	±	4.75	74.56	±	7.62
	(0.01	±	0)	(0.27	±	0.07)	(3.38	±	0.59)	(3.42	±	0.32)	(4.61	±	0.47)
Coffee and Tea^6^	0.00	±	0.00	0.16	±	0.16	3.21	±	1.29	3.37	±	0.61	3.20	±	0.73
	(0	±	0)	(0.02	±	0.02)	(0.26	±	0.10)	(0.23	±	0.04)	(0.20	±	0.05)

Data based on Day 1 dietary recalls

SEM, Standard Error of the Mean. 1) 100% Juice includes: citrus, apple, other fruit, vegetable, baby juice. 2) Water includes: tap, bottled, flavored, carbonated, enhanced, fortified, baby water. 3) Milk includes: milk, flavored milk, milk shakes, dairy drinks, milk substitutes. 4) Diet Beverages includes: diet soft drinks, diet sport drinks, diet energy drinks, other diet beverages. 5) Sugar Sweetened Beverages includes: soft drinks, fruit drinks, sport drinks, energy drinks, nutritional beverages. 6) Coffee and Tea includes: coffee, tea

### Nutrient intakes

Table 7 shows the percent of children below the Estimated Average Requirement (EAR) or above the AI for under-consumed nutrients that pose a public health concern by age. Over 90% of all children 2–3 and 4–5 years of age consumed less than the EAR for vitamin D. From 1 to 5 years our data showed the percent of children who consumed calcium amounts below the EAR consistently increased. Nearly 33% of children 4 to 5 years had calcium intakes below the EAR. Like calcium and vitamin D, potassium intake also decreased with age, particularly after the first year. Less than 1% of children had potassium intakes above the AI by 4 to 5 years of age.

**Table 7:** Percent of children below the Estimated Average Requirement (EAR) or above the Adequate Intake (AI) by age, NHANES 2011–2014

	12–23 months (*n* = 397)				2–3 years (*n* = 839)				4–5 years (*n* = 675)			
	% > AI	% < EAR		SEM	% > AI	% < EAR		SEM	% > AI	% < EAR		SEM
Calcium (mg)		2.22^a^	±	0.75		2.90^a^	±	0.76		32.74^b^	±	3.13
Iron (mg)		1.67^a^	±	0.58		0.75^a^	±	0.19		2.37^ab^	±	0.45
Magnesium (mg)		0.02^a^	±	0.03		0.004^a^	±	0.01		1.15^b^	±	0.36
Phosphorus (mg)		0.03	±	0.05		0.01	±	0.01		0.02	±	0.02
Vitamin A, (mcg)		0.69^a^	±	0.34		0.63^a^	±	0.28		3.88^b^	±	1.06
Vitamin C (mg)		0.39	±	0.21		0.16	±	0.09		1.96	±	0.73
Vitamin D (mcg)		76.53^a^	±	3.51		90.01^b^	±	1.96		93.78^b^	±	1.67
Zinc (mg)		0.02	±	0.02		0.00	±	0.02		0.45	±	0.32
Potassium (mg)	1.67		±	0.70	2.82		±	0.68	0.18		±	0.12

Data from usual intake using the National Cancer Institute method with two days of dietary recalls. Nutrients from breast milk are not be included in these analyses

EAR, Estimated Average Requirement; AI, Adequate Intake; SEM, Standard Error of the Mean. ^a,b^ Means sharing the same superscripts for EAR percentages after 1 year are not significantly different across ages, *p* < 0.01

## Discussion

Authoritative sources have put forward dietary recommendations, including for beverages, to help populations maintain health and reduce the risk for disease [3, 12, 14]. Traditionally the DGAs have only provided dietary recommendations for Americans ages 2 years and older; however, the 2020–2025 DGAs will include recommendations for infants and children from birth to 2 years [7]. As such, the USDA and HHS have asked for additional data on dietary and beverage patterns for this young age group [6]. Our study utilized NHANES data to determine if beverage recommendations were followed for American children during their first 5 years of life. Our results indicate that beverage recommendations were generally not followed across early childhood, despite providing significant amounts of kcals to the diet.

From birth to 6 months the AAP recommends exclusive breastfeeding or formula feeding [12], which the IOM explains as no supplementation of water, juice, nonhuman milk, or foods [27]. However, our analysis showed that on any given day nearly 12% of infants under 6 months consumed 100% juice and over 21% consumed water. Early introduction of water to infants less than 6 months may be of concern since it can displace consumption of nutrient-rich human milk or infant formula.

### Milk

Our results show that milk was introduced earlier than recommended. On any given day, nearly 22% of children 6 to 11 months already consumed cow’s milk in quantities ranging from 0.2 to 0.4 cups per day. The mean amount of milk consumed on any given day decreased after 2 years for each of the race/ethnic groups analyzed. All groups consumed less than 2 cups of milk per day among children 2 to 5 years, with Non-Hispanic Black children consuming the least. A decrease in milk consumption as children age has been reported previously in population, survey, and clinical trial studies [22, 28, 29]. Differences among race/ethnic groups have been reported for milk consumption among children and adolescents (2–18 years) based on 2001–2004 NHANES data [21]. These results, along with our current data, highlight that across all ages a very small proportion of Non-Hispanic Black children consume sufficient amounts of milk. Cross-sectional data indicate that diet quality can be related to socioeconomic status [30]; however, factors such as income and education level were not included in in this study. Additionally, NHANES 2013–2014 data show that total dairy and milk consumption do not vary by family income [31]. Differences in milk consumption might be explained by genetic predisposition of Non-Hispanic Blacks toward lactose intolerance, as well as cultural beliefs and perceptions about consumption of dairy products [32, 33]. Previous research indicates that approximately 20% of Hispanic, Asian, and Non-Hispanic Black children younger than 5 years of age have evidence of lactase deficiency and lactose malabsorption [34]. Nevertheless, the AAP encourages children with lactose intolerance to keep dairy foods in the diet to help meet nutrient needs [35]. Thus, personalized management strategies can help, such as consuming small amounts of dairy foods at a time, consuming milk with meals, and opting for lactose-free or reduced-lactose cow’s milk to help ensure children meet nutrient recommendations [36]. Our results show that the Asian population, who is believed to have a high prevalence of lactase deficiency as well [35], has seemingly overcome these barriers as statistically Asian children consumed the same amount of milk as Non-Hispanic White and Hispanic children from 2 to 5 years.

### 100% juice

Our results show that 100% juice consumption decreased after 2 to 3 years, similar to milk consumption. The DGA recognizes 100% juice as a beverage that can contribute beneficial nutrients to the diet, but states that juice should not provide more than half of the recommended fruit intake [3]. One-hundred percent juice can provide substantial amounts of vitamin C and potassium, but lacks the dietary fiber found in whole fruits or vegetables [20]. Thus, the AAP recommends that juice should not be introduced into the diet of infants before 12 months of age, and the intake of juice should be limited to 4 oz. or 4–6 oz. per day for children 1 to 3 years and 4 to 6 years, respectively [11]. Based on these recommendations, our results indicate that on any given day, children consumed 100% juice too early (over 10% of infants 0–5 months and nearly 40% of 6–11 month old infants) and that Non-Hispanic Black children consumed the most 100% juice under 2 years of age. Non-Hispanic White, Non-Hispanic Black, Hispanic, and Asian children 2 to 3 years all consumed amounts of 100% juice that exceeded the AAP recommendations [11]. Higher 100% juice consumption for Non-Hispanic Black children has been reported previously [20]. Experts caution that excessive juice consumption may be associated with malnutrition, tooth decay, excessive weight gain, or gastrointestinal discomfort [11, 37–39].

### Sugar sweetened beverages

SSB consumption steadily increased over time, consistent with prior research [29]. Regardless of race/ethnicity, we found an increase in consumption of SSBs as children got older and by 4 to 5 years, on any given day, over 51% of all children consumed SSBs. Increased consumption of SSBs by children is of concern due to their contribution to empty kcals and added sugar intake as well as displacement of more nutrient-rich beverages such as milk and 100% juice in the diet. Prior research showed increased consumption of SSBs in children was associated with an overall decrease in children’s diet quality [17], an increase in childhood obesity [40], and increased risk for development of non-communicable diseases [18]. A recent scientific statement by the American Heart Association indicates that there is strong evidence that added sugar intake among children is associated with increased cardiovascular disease risk through increased energy intake, increased adiposity, and dyslipidemia. It was also recognized that when added sugars are consumed within energy limits, it is better if the sugars are consumed as part of nutrient-rich foods [18].

Consumption of SSB in this sample varied by race/ethnicity. Non-Hispanic Black children consumed the most SSBs during 0–23 months. During these first 2 years of life, Non-Hispanic Black children consumed nearly 2, 3, and 6 times more SSB than Hispanic, Non-Hispanic White, and Asian children, respectively. These results align with previously reported data [15, 41]. Garnett et al. surveyed parents on their child’s beverage intake and found that Non-Hispanic Black children had the highest consumption of SSBs for all age groups. Moreover, Hispanic and Non-Hispanic Black mothers were more likely to report that their 2 yr. old children consumed a SSB at least once a week [15].

### Nutrients

After 1 year, milk provided the most kcals of the beverages examined. This is appropriate as milk a nutrient rich food providing essential nutrients associated with proper growth [12], and its consumption has been associated with improved diet quality and bone health among children [42]. Milk is the number one food source of nine essential nutrients for children; protein, calcium, potassium, phosphorus, vitamins A, D, B12, riboflavin and niacin as niacin equivalents [5]. Among beverages consumed by American infants and children 0 to 5 years of age, our results show milk provided the largest nutrient contribution.

Non-Hispanic Black children consumed the least amounts of calcium and vitamin D from 1 to 5 years. Over 25% and 95% of Non-Hispanic Black children 1–5 years of age had inadequate intakes of calcium and vitamin D, respectively, as compared to 8.2 and 83.2% of Hispanics and 12.9 and 88.5% for Non-Hispanic White children (data not shown). Non-Hispanic Black children were the only race/ethnic group who consumed more SSBs than milk at 4 to 5 years of age. Regardless of race/ethnicity, our data showed that the decreased consumption of nutrient-rich beverages, such as milk, as children got older was reflected in decreased intakes of the nutrients of public health concern. For children 4 to 5 years, nearly 94% had vitamin D intakes below the EAR, over 32% had calcium intakes below the EAR and less than 1 % had potassium intakes above the AI. These low intakes for the under-consumed nutrients of public health concern among young American children are alarming as they are necessary for normal growth and development [36].

### Strengths and limitations

One of the strengths of this study was the use of a sample representative of the U. S population. A second strength was the inclusion of infants and children 0 to 24 months age group as many previous reports have focused only on children 2+ years. Finally, we utilized the National Cancer Institute method for estimating usual intake to estimate percentage of the population meeting or below dietary recommendations. This study was limited by the fact that we were not able to analyze race/ethnic data in smaller age sub-groups due to the small sample sizes among young infants (0–12 months). Additionally, due to the fact that NHANES data dietary information may be subject to over- or under-reporting and the accuracy of parents/caregivers reporting of their children’s food intake is unknown.

## Conclusions

Beverage consumption among American infants and children 0 to 5 years, as reported in NHANES 2011 to 2014, did not consistently follow recommendations from authoritative sources. Consumption of milk and 100% juice decreased with age, particularly after 2 years, while consumption of SSBs steadily increased. Simultaneously, as children got older they had lower intakes of under-consumed nutrients of public health concern; calcium, vitamin D and potassium. Differences in beverage intakes exists when comparing various race/ethnic groups. Non-Hispanic Black children consumed the most 100% juice from 2 to 3 years, exceeding recommended amounts, and throughout all age groups they consumed the least milk and most SSBs. These results highlight that improving beverage consumption patterns, particularly for Non-Hispanic Black children, could help improve overall diet quality that can reduce risk for obesity and other diet-related chronic diseases.

## Abbreviations

AAP: American Academy of Pediatrics
AI: Adequate Intake
AND: Academy of Nutrition and Dietetics
BBR: Balanced repeated replicates
DGA: Dietary Guidelines for Americans
EAR: Estimated Average Requirement
HHS: Health and Human Services
IOM: Institute of Medicine
kcals: Kilocalories
NCI: National Cancer Institute
NHANES: National Health and Nutrition Examination Survey
oz: Ounces
SE: Standard error
SSB: Sugar sweetened beverages
USDA: United States Department of Agriculture
